# Assessing pharmacists and other healthcare providers’ knowledge of hand sanitization during COVID-19 pandemic in Jordan: A comparative study

**DOI:** 10.1371/journal.pone.0283328

**Published:** 2023-04-18

**Authors:** Rula M. Darwish, Mohammad AlMasri, Khawla Ammar, Rama AlMasri, Hani Al- Najar, Mahmoud M. Al-Masri

**Affiliations:** 1 Department of Pharmaceutics and Pharmaceutical Technology, School of Pharmacy, The University of Jordan, Amman, Jordan; 2 School of Medicine, The University of Jordan, Amman, Jordan; 3 King Hussein Cancer Centre, Survey Unit, Amman, Jordan; 4 Department of Surgery King Hussein Cancer Centre, Amman, Jordan; Zarqa University, JORDAN

## Abstract

**Background:**

Health care providers including pharmacists are often on the first line when dealing with COVID -19; they can be under threat of contracting and spreading the disease. We aimed to assess and compare their knowledge of hand sanitization during COVID-19 pandemic to improve quality of care.

**Methods:**

A cross-sectional study was conducted in Jordan, on healthcare providers in different settings from 27 October till 3 December 2020, using a pre-validated electronic questionnaire. Participants (n = 523) were healthcare providers practicing in different settings. Descriptive and association statistical analyses were produced on the data using SPSS 26. Chi square was used for the categorical variables, and One way ANOVA was used on the continuous and categorical variables.

**Results:**

A significant difference was recorded in total knowledge mean according to gender (59.78 vs 61.79 p = 0.030) in favor of men, and between pharmacists and other healthcare providers in favor of the latter (59.22 vs 61.45, p = 0.02). No significant difference was generally noticed between those who attended hand hygiene training and those who did not.

**Conclusion:**

Healthcare providers’ knowledge of hand hygiene was generally good among participants, regardless of training and it was possibly increased because of fear of COVID-19 infection. Physicians were the most knowledgeable in regard of hand hygiene while pharmacists were the least among healthcare providers. Thus, structured, more frequent, and tailored training on hand sanitization in addition to new educational strategies are recommended for healthcare providers, in particular, pharmacists for better quality of care especially in pandemics.

## Introduction

COVID-19 has dramatically affected the globe and imposed great stress and responsibility on healthcare providers. Evidence shows that this virus is transmitted through respiratory droplets and close contact with an affected individual or contaminated object [1]. Infected persons can transmit the virus even when they have no or only mild symptoms [2]. Consequently, preventing the spread is extremely important to reduce the general burden of the disease. Healthcare providers, licensed persons that provides healthcare services (HCPs), who are in close contact with COVID-19 patients are at increased risk of acquiring the infection as well as transferring it; therefore, they are required to protect themselves and prevent transmission of this disease in their healthcare settings. Hand sanitization is the act of physically or mechanically eliminating dirt, organic matter, and/or microbes from one’s hands. It includes the use of alcohol-based hand sanitizer and/or hand washing with soap and water and although simple it is an effective way to prevent the spread of COVID-19 in healthcare settings [3]. World Health Organization (WHO) recommends hand washing with soap and water only when visibly soiled while alcohol rub is recommended in most clinical situations. This might be because alcohol rub (usually 60% ethanol or 70% isopropanol) has a wide spectrum of activity and is cost effective, accessible at the point of care, and has better compliance [4–6]. Nevertheless, many studies have shown that HCP compliance with hand hygiene is generally low in both developing and developed countries [7–11]. This might be due to several factors, including overcrowding, lack of time, irritation caused by hand hygiene products, lack of sufficient knowledge, and lack of a positive attitude to the best practices of hand hygiene [12, 13].Only few studies have dealt with pharmacists and hand hygiene [14, 15]. This work is a cross sectional study that aims to evaluate and compare, whenever appropriate, the healthcare providers’ knowledge of hand sanitization and its usage in the different professional healthcare settings during the COVID-19 pandemic in Jordan. The study addresses different variables identified in previous work in an attempt to improve quality of care[12, 13, 16]. This would help to tailor intervention to promote more appropriate use of hand sanitizers.

## Methods

### Study context and survey tool

#### Study population and study setting

Five hundred twenty-three healthcare providers from different healthcare settings participated in the study.

Every HCP in any healthcare institution was a potential participant, students were not targeted and we did not receive responses from any student. The sample size was estimated based on Draugalis and Plaza [17] reference, where 381 responses at least were needed, but we were able to reach more than 500 respondents.

### Data collection

A cross-sectional, pre-validated structured and anonymous online survey in English language, was administered to HCPs. The standard tool of the WHO questionnaire on hand sanitization was used (supplementary material) [18]. The questionnaire was uploaded via Survey Monkey. It was tested, and circulated among different groups that included health care providers from different sectors on WhatsApp, Facebook, healthcare facility homepages and emails. The link was generated, and sent to the healthcare facility staff and they were asked to share the link with the members. The tool contains nine multiple choice questions, twelve yes/no questions, four true/ false questions. In total the score is measured out of 25, one point was given to the correct answer while the incorrect answer was given zero when calculating the total knowledge scores. A score out of 100 was calculated and grouped according to the part mentioned in the methods section (Data collection and analysis). The data collection was conducted from 27 October till 3 December 2020. Participants were able to complete the survey only once. The survey was anonymous and confidential. An introductory paragraph outlining the purpose of the study was posted along with the survey. Missing data were evaluated and up to 5% missing parts was acceptable.

The questionnaire collected demographic information about the participants including age, sex, sector, level of education, and previous training on hand hygiene within the last three years.

### Data analysis

Data were analyzed using SPSS 26. Demographic data were described using frequencies and percentages. Analysis of correct answers for each question was done, and their significance was reported by both chi square in case of categorical data and one-way ANOVA in case of continuous data. P values of ≤ 0.05 were considered significant. The total mean knowledge scores were calculated, the total number of questions were 25, the score was calculated out of 100. and those who scored > 75% were grouped as adequate knowledge, 50%-74% moderate knowledge, and less than 50% insufficient knowledge [19]. Only those who answered all knowledge questions had mean knowledge scores. The knowledge scores were compared based on different variables including: gender, age groups, profession in general then pharmacists vs other HCP. Participants were classified as previously attended hand hygiene training and did not attend and knowledge scores were compared based on this to measure the importance of training in knowledge improvement. The STROBE cross sectional reporting guidelines was used [20].

### Ethical consideration

The Ethics and Research Committee of an area hospital (number 20 / 167) approved the research. The study targeted HCPs, above the age of 18, no minors were included, no written informed consent for participation was not required. Participants had the study information in the cover page and were informed that participation was voluntary and they had the right not to complete the survey or withdraw at any time. Completing the survey was considered as consent from the participants.

## Results

### Demographic characteristics and hand hygiene training

A total of 523 questionnaires were adequately filled by online survey. Among the participants, pharmacists were 196 (37.5%), physicians were 125 (23.9%), nurses were 111 (21.2%) and paramedics were 91 (17.4%). There were 207 participants (42.2%) in the age group 25–35, and the mean age was 29.4 years (Table 1). The number of participants working in the private sector was 195 (42.7%), public sector 115 (25.2%), and educational institutes110 (24.1%). The majority held Bachelor degrees (N = 335; 64.5%). More than half of them (N = 314; 60.2%) got training on hand hygiene within the last three years (physicians N = 76, nurses N = 88, pharmacists N = 92, and paramedics N = 58).

**Table 1:** Demographics and characteristic of the healthcare providers.

		Total	Pharmacist		Other healthcare worker			Frequency of missing data
			N = 196		N = 326			
			Frequency	Percentage	Frequency	Percentage	P value	
Gender	Female	351	153	78.1%	198	60.6%	<0.001	zero
	Male	172	43	21.9	129	39.4%		
Age	19–24	187	113	57.7%	74	25.2%	<0.001	33
	25–35	207	45	23%	162	55.1%		
	Above 35	96	38	19.4%	58	19.7%		
Sector	Public	115	41	21.5%	74	27.8%	0.001	66
	Private	195	91	47.6%	104	39.1%		
	Educational institute	110	35	18.3%	75	28.2%		
	Others	37	24	12.6%	13	4.9%		
Educational level	Diploma	50	18	9.2%	32	9.9%	<0.001	4
	Bachelor	335	148	75.9%	187	57.7%		
	Graduated studies	134	29	14.9%	105	32.4%		
Attend training	Yes	314	92	46.9%	222	68.1%	<0.001	1
	No	208	104	53.1%	104	31.9%		

Chi square test was used to assess level of associations

Table 2 shows that the mean knowledge score was associated with gender (p = 0.030) and profession (p = 0.047) but not with age (p = 0.361) or sector (p = 0.806). The pharmacist knowledge score was significantly lower than other healthcare providers (p = 0.02). The average knowledge score was 60.4%. Those who received training scored higher than those who did not (mean 61.4 vs 59.0, p = 0.003.

**Table 2:** Total knowledge mean base on gender, age group, sector, and profession.

	Knowledge				
		Mean			P value
Gender	Female	59.78	314	9.10	0.030
	Male	61.788	148	9.50	
	Total		462		
Age Groups	19–24	59.67	170	10.01	0.361
	25–35	60.89	185	8.96	
	Above 35	61.14	79	8.73	
	Total		434		
Sector	Public	59.85	102	9.38	0.806
	Private	60.15	175	8.86	
	Educational	60.69	99	9.99	
	Total		376		
Profession	Nurses	60.87	96	8.44	0.047
	Pharmacist	59.22	169	9.74	
	Physicians	61.95	113	9.03	
	Total		378		
Pharmacist vs others	Pharmacist	59.22	169	9.74	0.02
	Physicians/ nurses	61.45	209	8.76	
	Total		378		
Attend training	Yes	61.37	278	8.99	0.003
	No	58.98	184	9.50	
	Total		462		

One-way ANOVA test was used

### Knowledge of respondents about hand hygiene

Table 3 shows that a large proportion of the participants (N = 471; 92.3%) knew the importance of hand hygiene before touching patients to prevent transmitting germs while only 51.2% (N = 268) knew that hands are the main route of cross-transmission of potentially harmful germs between patients in a health-care facility. From these, 86 (32.1%) were physicians, 68 (25.4%) pharmacists, and 66 (24.6%) nurses (p value ≤ 0.001). Only 31.3% (N = 163) considered germs already present on or within the patient as the most frequent source of germs responsible for healthcare-associated infections. A substantial number of participants (N = 468; 91.3%) were not knowledgeable about the importance of hand hygiene actions in preventing the transmission of germs to the patient immediately after a body fluid exposure. Almost the same number (N = 470; 92.4%) were not aware of the role of hand hygiene actions in preventing the transmission of germs to patients after exposure to their immediate surroundings. A total number of (N = 466, 91.9%) () acknowledged the importance of hand hygiene in preventing the transmission of germs to patients, immediately before a clean/aseptic procedure.

**Table 3:** Knowledgeable and not knowledgeable participants in regard of statements of hand hygiene at the level of whole sample.

Statement about Hand Hygiene	Knowledgeable	Not knowledgeable	Missing data frequency
Main route of cross-transmission of potentially harmful germs between patients in a health-care facility, healthcare workers hand when not clean	268 (51.2%)	255 (48.8%)	zero
Most frequent source of germs responsible for healthcare-associated infections germs already present on or within the patient	163 (31.3%)	360 (68.7%)	zero
Hand hygiene actions prevent transmission of germs to the patient before touching a patient	471 (92.3%)	39 (7.7%)	13
Hand hygiene actions prevent transmission of germs to the patient immediately after a risk of body fluid exposure.	44 (8.7%)	468 (91.3%)	11
Hand hygiene actions prevent transmission of germs to the patient after exposure to the immediate surroundings of a patient	38 (7.6%)	470 (92.4%)	15
Hand hygiene actions prevent transmission of germs to the patient immediately before a clean/aseptic procedure	466(91.9%)	41 (8.1%)	16

*knowledgeable, answered correctly

Table 4 shows a significant difference between the knowledge of pharmacists and other HCPs in the major aspects of hand hygiene including knowing that their hands are the main route of cross-transmission when not clean (p < 0.001), hand hygiene actions prevent transmission of germs to the HCPs after touching a patient (p = 0.023) and importance of hands in preventing transmission of germs to the HCPs immediately after body fluid exposure (p = 0.013). In addition to the importance of hand hygiene before palpation of the abdomen (p <0.001), and the association of wearing jewelry and artificial fingernails with the increased likelihood of colonization of hands with harmful germs (p = 0.003 and <0.001, respectively). On the other hand, pharmacists were significantly more knowledgeable in other aspects of hand hygiene such as the importance of hand hygiene actions after exposure to the immediate surroundings of a patient in preventing transmission of germs (p = 0.046) and the preference of hand rubbing over handwashing (p = 0.012).

**Table 4:** Difference between knowledge pharmacists and other healthcare worker in certain areas of hand hygiene.

Statement about Hand Hygiene	Profession	Knowledgeable	Not knowledgeable	p-value
**Main route of cross-transmission of potentially harmful germs between patients in a health-care facility, healthcare workers hand when not clean**	Pharmacist	68 (34.7%)	128 (65.3%)	<0.001
	196			
	Other healthcare worker	200 (61.5%)	125 (38.5%)	
	325			
**Hand hygiene actions prevent transmission of germs to the patient before touching a patient**	Pharmacist	168 (88.9%)	21 (11.1%)	0.034
	189			
	Other healthcare worker	303 (94.1%)	19 (5.9%)	
	322			
**Hand hygiene actions prevent transmission of germs to the patient after exposure to the immediate surroundings of a patient**	Pharmacist	20 (10.5%)	171 (89.5%)	0.046
	191			
	Other healthcare worker	18 (5.7%)	300 (94.3%)	
	318			
**Hand hygiene actions prevent transmission of germs to the healthcare worker after touching a patient**	Pharmacist	169 (91.4%)	16 (8.6%)	0.023
	185			
	Other healthcare worker	304 (96.2%)	12 (3.8%)	
	316			
**Hand hygiene actions prevent transmission of germs to the healthcare worker immediately after a risk of body fluid exposure**	Pharmacist	168 (90.8%)	17 (9.2%)	0.013
	185			
	Other healthcare worker	304 (96.2%)	12 (3.8%)	
	316			
**Hand rubbing is more effective against germs than handwashing**	Pharmacist	77 (40.7%)	112 (59.3%)	0.012
	189			
	Other healthcare worker	95 (29.9%)	223 (70.1%)	
	318			
**Which type of hand hygiene method is required in the following situations?**	Pharmacist	90 (46.9%)	102 (53.1%)	<0.001
	192			
	Other healthcare worker	207 (63.7%)	118 (36.3%)	
**Before palpation of the abdomen: Rubbing**				
	325			
**Which type of hand hygiene method is required in the following situations?**	Pharmacist	56 (28.9%)	138 (71.1%)	0.004
	194			
	Other healthcare worker	58 (17.9%)	266 (82.1%)	
**After emptying a bedpan: Rubbing**				
	324			
**Which of the following should be avoided, as associated with increased likelihood of colonisation of hands with harmful germs?**	Pharmacist	159 (82%)	35 (18%)	0.003
	194			
	Other healthcare worker	294 (90.7%)	30 (9.3%)	
**Wearing jewelry**	324			
**Which of the following should be avoided, as associated with increased likelihood of colonisation of hands with harmful germs?**	Pharmacist	161 (83%)	33 (17%)	<0.001
	194			
	Other healthcare worker	308 (95.4%)	15 (4.6%)	
**Artificial fingernails**	323			

Chi square test was used in this table

Table 5 shows that generally all the participants were aware of hand hygiene practices regardless whether they received training on hand hygiene or not. The significance between trained and untrained personnel was only noticed in the knowledge of the main route of cross-transmission of potentially harmful germs between patients in a healthcare facility (p = 0.012), importance of hand hygiene after touching a patient (p = 0.052), minimal time needed for alcohol-based hand rub to kill most germs on the hands (p = 0.006), and effect of wearing jewelry and artificial fingernails on hand hygiene (p = 0.005 and 0.002, respectively).

**Table 5:** Knowledge variation between participants who attended training and who did not attend training on hand hygiene per questions.

		*Correct response		
Question	Correct answer	Attend Training 313	Did Not attend training 208	p-value
**Which of the following is the main route of cross-transmission of potentially harmful germs between patients in a health-care facility, (healthcare workers’ hands when not clean)**	Healthcare workers hands when not clean	175 (55.9%)	93 (44.7%)	0.012
**What is the most frequent source of germs responsible for healthcare associated infections**	Germs already present on or within the patient	102 (32.7%)	61(29.3%)	0.418
** Which of the following hand hygiene actions prevent transmission of germs to the patient? Before touching a patient**	Yes	284 (92.8%)	187(91.2%)	0.512
** A. Immediately after a risk of body fluid exposure**	No	28 (9.1%)	16 (7.8%)	0.591
** B. After exposure to the immediate surroundings of a patient**	No	22 (7.3%)	16 (7.8%)	0.831
** C. Immediately before a clean/aseptic procedure**	Yes	284 (93.4%)	182(89.7%)	0.128
**Which of the following hand hygiene actions prevent transmission of germs to the health-care worker?**				
** A. After touching a patient**	Yes	290 (96%)	183 (92%)	0.052
** B. Immediately after a risk of body fluid exposure**	Yes	289 (95.1%)	183 (92.9%)	0.309
** C. Immediately before a clean/aseptic procedure**	Yes	234 (77.7%)	155 (79.1%)	0.723
** D. After exposure to the immediate surroundings of a patient**	No	14 (4.6%)	13 (6.7%)	0.325
**Which of the following statements on alcohol-based hand rub and handwashing with soap and water are true?**				
** A. Hand rubbing is more rapid for hand cleansing than handwashing**	True	210 (68.9%)	134 (66.3%)	0.553
** B. Hand rubbing causes skin dryness more than handwashing**	False	243 (79.2%)	155 (75.6%)	0.345
** C. Hand rubbing is more effective against germs than handwashing**	True	97 (31.9%)	75 (36.9%)	0.240
** D. Handwashing and hand rubbing are recommended to be performed in sequence**	False	96 (31.3%)	54 (26.2%)	0.217
** What minimal time is needed for alcohol-based hand rub to kill most germs on your hands?**	20 seconds	217 (69.3%)	120 (57.7%)	0.006
**Which type of hand hygiene method is required in the following situations?**				
** A. Before palpation of the abdomen**	Rubbing	189 (60.8%)	108 (52.4%)	0.06
** B. Before giving an injection**	Rubbing	196 (62.6%)	113(54.9%)	0.078
** C. After emptying a bedpan**	Rubbing	67 (21.5%)	47 (22.8%)	0.718
** D. After making a patient’s bed**	Rubbing	97 (31.3%)	71 (34.3%)	0.474
**Which of the following should be avoided, as associated with increased likelihood of colonisation of hands with harmful germs?**				
** A. Wearing jewelry**	Yes	284 (90.7%)	169 (82.4%)	0.005
** B. Damaged skin**	Yes	286 (92.6%)	190 (92.7%)	0.957
** C. Artificial fingernails**	Yes	291 (93.9%)	178 (86%)	0.002
** D. Regular use of a hand cream**	No	186 (60.4%)	115 (56.7%)	0.401

*The total for those who attended training varies between 301 and 313 for the questions, and between 195 and 208 for those who did not attend.

## Discussion

The exact contribution of hand hygiene to the reduction of direct and indirect spread of coronaviruses between people is currently unknown, yet hand washing removes pathogens, and laboratory data showed that formulations including alcohol in certain concentrations recommended by the Centers for Disease Control and Prevention (CDC) inactivate severe acute respiratory syndrome coronavirus 2 (SARS-CoV-2) [21]. Knowledge of good hand hygiene according to WHO guidelines among healthcare providers is vital for lowering the risk of health-care associated infections [22].

Most of the participants in this study were young (< 35) with no significant effect on the knowledge mean score. Age groups can vary from one study to another and this could reflect on the level of adherence to hand hygiene [19, 23]. A healthcare provider’s age can be correlated to the number of years of practice, and studies have indicated that age and experience are possibly positively correlated with adherence to hand hygiene [24]. However, it didn’t show any significant difference in this study. This might indicate the need to improve knowledge on hand hygiene through implementing educational sessions targeting fresh graduates as well as practicing HCPs. The findings showed a significant difference in gender with more males having knowledge of hand hygiene. This was not in concordance with other studies which showed no difference in the knowledge between males and females [25, 26].

Hand hygiene training was found to have an association with knowledge levels of hand hygiene. Our findings showed that a significant difference between trained and untrained heath care providers in the total knowledge mean this necessitates a revision of the training provided in the aim of improving the outcomes.

The results show a significant difference in knowledge among HCPs, with pharmacists having the lowest knowledge score. This is in concordance with a study by Chee Sheong et al. [27] which showed poor knowledge of pharmacists on hand hygiene regardless of training. Physicians, on the other hand, had the highest and these results were in concordance with a study in Emirates on healthcare professionals’ hand hygiene where it showed that physicians’ hand hygiene knowledge was slightly higher than that of nurses (78.5% versus 73.5%) [28]. However, the findings contradict other studies which revealed that nurses knowledge on hand hygiene practices was better than physicians [29, 30]. Consequently, the outcomes dictate that weaknesses in knowledge regarding hand hygiene among pharmacists should be addressed to ensure better quality service. It is possible that this variation in knowledge is due to the need for direct contact of nurses and physicians with patients unlike pharmacists who are usually responsible for determining whether the prescribed medications are optimally meeting the patient’s needs and goals of care.

Furthermore, this study showed that different professions have different knowledge strengths. Physicians were significantly more knowledgeable about the role of hands as the main route of cross-transmission, performing handwashing and hand rubbing in sequence, and the effect of jewelry and artificial fingernails as a source of infection while nurses were significantly more knowledgeable about the effect of rubbing causing skin dryness. Pharmacists were significantly more knowledgeable about the importance of hand hygiene after exposure to the immediate patient’s surroundings and body fluid. The results of the current study are also inconsistent with findings of other studies on the knowledge of hand washing across professional groups and by workplaces [31, 32]. One of the possible explanations for these findings is that different degrees of contact of heath care providers with patients and their body fluids is associated with their knowledge of the risk of infection and thus their level of awareness and knowledge of hand hygiene [32]. These different knowledge strengths among different professions may suggest different training programs that focuses on the nature of each profession, without neglecting the major principles of hand hygiene.

The results showed no differences between public, private, and educational settings among different professions in relation to knowledge on different aspects of hand hygiene. In this context, a study done in Gambia [33] showed that private healthcare settings provided better hand hygiene resources for their HCPs. However, neither private nor public healthcare settings provided satisfactory hand hygiene training and advice on hand hygiene performances to their HCPs. The findings are in agreement with other studies in which training had no effect on knowledge level. A study by Duggan et al. even showed a negative relationship between professional education and the compliance of hand washing [30]. On the other hand, a study by Suchitra et al. revealed that education had a positive impact on the retention of knowledge among all healthcare provider groups [34]. Therefore, it is important to revise the training programs provided in order to improve knowledge which would be reflected on the application.

### Strengths and limitations

The diversity of HCPs in different healthcare settings in this study should produce a rich evidence based outcome which allows exploration of the reasons for poor application of proper hand sanitization and thus develop strategies to promote the proper use of hand sanitizers and reduce risks associated with pandemics. The study, however, like any other cross-sectional design study, encountered difficulty in having a representative sample. Thus, the sample was chosen to be larger than the minimum needed size to be representative as much as possible. Another limitation was the lack of the geographical information. In spite of limitations, the results of this study, can be indicative, as it was conducted in the peak of the pandemic, where online communication was the most accessible used method and became familiar among different groups.

## Conclusion

This study highlighted gaps in knowledge among HCPs. Knowledge of hand hygiene was generally good among participants, regardless of training and possibly increased because of fear of COVID-19 infection. Physicians were the most knowledgeable in regard of hand hygiene while pharmacists were the least among healthcare providers.

## Recommendations

The findings emphasize the need to improve the quality of education and training to enable HCPs to deal with different infectious situations and emergency cases, such as COVID -19 pandemic, and thus ensure better quality of care delivery. The results indicate the need for structured, tailored, more frequent, and regularly re-evaluated hand hygiene training courses together with educational programs to be provided to HCPs. This would eventually translate into a behavioral change of attitudes and practices that would help in reducing the incidence of infections. Further policies and plans for follow up on this issue is always needed to improve quality of care. It is critical to improve the preparedness and awareness of HCPs to the important measures for prevention which would lead to improving the quality of care.

## Supporting information

S1 Data (XLSX)

S1 File (DOC)
